# Synthesis and Anticancer Activity of Some Novel Tetralin-6-yl-pyrazoline, 2-Thioxopyrimidine, 2-Oxopyridine, 2-Thioxo-pyridine and 2-Iminopyridine Derivatives

**DOI:** 10.3390/molecules16043410

**Published:** 2011-04-20

**Authors:** Ebtehal S. Al-Abdullah

**Affiliations:** Department of Pharmaceutical Chemistry, College of Pharmacy, King Saud University, Riyadh 11451, Saudi Arabia; E-Mail: ealabdullah@ksu.edu.sa

**Keywords:** tetrahydronaphthalenes, 2-pyrazoline, 2-thioxopyrimidine, 2-oxopyridine, 2-thioxopyridine, 2-iminipyridine, anticancer activity

## Abstract

The title compounds were prepared by reaction of 6-acetyltetralin (**1**) with different aromatic aldehydes **2a-c**, namely 2,6-dichlorobenzaldehyde, 2,6-diflouro-benzaldehyde, and 3-ethoxy-4-hydroxybenzaldehyde, to yield the corresponding α,β-unsaturated ketones **3a-c**. Compound **3b** was reacted with hydrazine hydrate to yield the corresponding 2-pyrazoline **4**, while compounds **3a,b** reacted with thiourea to afford the 2-thioxopyrimidine derivatives **5a**,**b**, respectively. The reaction of **1**, and the aromatic aldehydes **2a-c** with ethyl cyanoacetate, 2-cyano-thioacetamide or malononitrile in the presence of ammonium acetate yielded the corresponding 2-oxopyridines **6a**,**b**, 2-thioxopyridines **7a-c** or 2-iminopyridines **8a**,**b**, respectively. The newly prepared compounds were evaluated for anticancer activity against two human tumor cell lines. Compound **3a** showed the highest potency with IC_50_ = 3.5 and 4.5 μg/mL against a cervix carcinoma cell line (Hela) and breast carcinoma cell line (MCF7), respectively.

## 1. Introduction

Cancer is presently responsible for about 25% of deaths in developed countries and for 15% of all deaths worldwide. It can therefore be considered as one of the foremost health problems, with about 1.45 million new cancer cases expected yearly. Antitumor chemotherapy is nowadays a very active field of research, and a huge amount of information on the topic is generated every year [1,2]. Although there is a large amount of information available dealing with clinical aspects of cancer chemotherapy, we felt that there was a clear need for an updated treatment from the point of view of medicinal chemistry and drug design [3]. Diverse chemotherapeutic activities were ascribed to substituted tetralin (tetrahydronaphthalene)-heterocycles [4,5] and tetrahydronaphthalene derivatives, especially those incorporated into heterocyclic systems [6]. It has been reported that this type of compounds possess a wide variety of biological activities including anti-HIV [5], antibacterial [4,7], hypotensive [8], antiarrythmic [9], molluscicidal [10], antiplatelet aggregation [11,12], anxiolytic and antidepressant [13,14] and anticancer [15,16] effects. Pyridin-2(1*H*)-ones are known to possess a range of biological activities such as analgesic, antifungal, antimalarial, antiinflammatory, antibacterial, anti-HIV, phytotoxic, antitumoral and antiviral properties [17,18,19,20,21,22,23,24]. Thus, it was of interest to synthesize some new heterocyclic derivatives carrying a tetrahydronaphthale moiety for evaluation as potential anticancer agents.

## 2. Results and Discussion

### 2.1. Chemistry

**Scheme 1:**
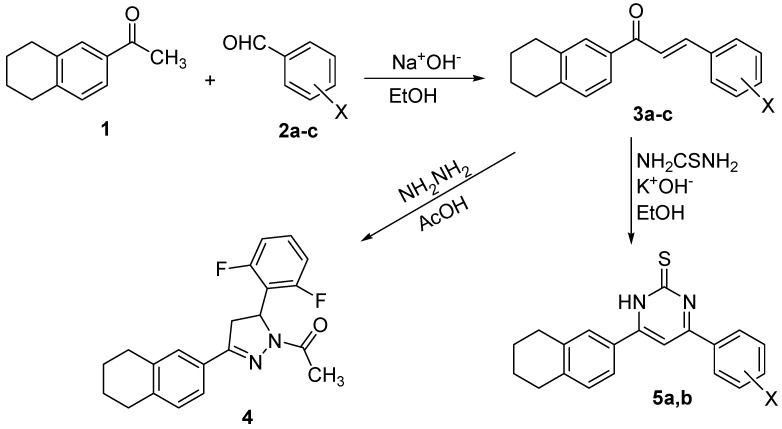
Synthetic pathways to compounds **3a-c**, **4** and **5a**,**b**.

α,β-Unsaturated ketons (chalcones) are active intermediates and excellent starting materials for the synthesis of several heterocyclic systems. Thus, a Claisen-Schmidt reaction of 1-(1,2,3,4-tetrahydro-naphthaline-6-yl)ethanone (**1**), prepared following the previously reported method of Allinger and Jones [25], with 2,6-dichlorobenzaldehyde (**2a**), 2,6-diflourobenzaldehyde (**2b**) or 3-ethoxy-4-hydroxybenzaldehyde (**2c**), in 10% ethanolic sodium hydroxide afforded the corresponding α,β-unsaturated ketons **3a-c** in 53, 42 and 80% yields, respectively. Condensation of the chalcone analogue **3b** with hydrazine hydrate in acetic acid yielded the corresponding 1-acetyl-2-pyrazoline derivative **4** in 92% yield. Meanwhile, the interaction of the chalcone analogues **3a**,**b** with thiourea in aqueous potassium hydroxide yielded the corresponding 2-thioxopyrimidines **5a**,**b** in 20 and 28% yields, respectively (Scheme 1, Table 1).

**Scheme 2:**
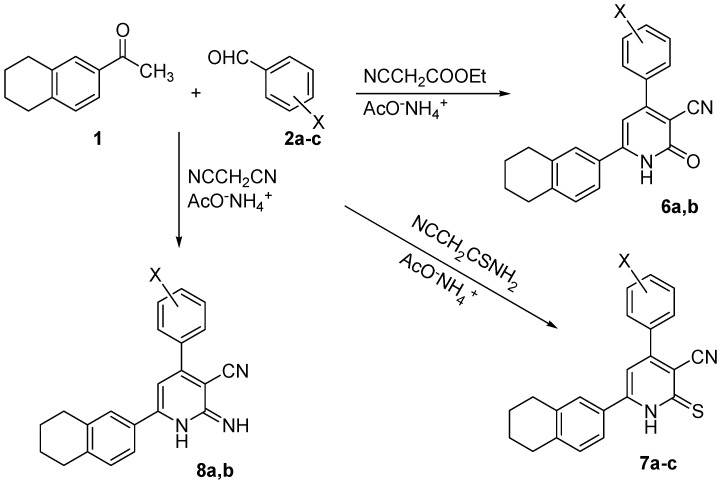
Synthetic pathways of compounds **6a**,**b**, **7a-c** and **8a**,**b**.

Condensation of compound **1** and the aromatic aldehydes **2a** or **2b** with ethyl cyanoacetate in presence of excess ammonium acetate in *n*-butanol afforded the corresponding 3-cyano-2-(1*H*)-oxopyridines **6a** and **6b** in a one pot reaction. Similarly, the reaction of compound **1** and the aromatic aldehydes **2a-c** with 2-cyanothioacetamide yielded the corresponding 3-cyano-2-(1*H*)-thioxopyridines **7a-c** in 25, 53 and 90% yields, respectively. The 3-cyano-2-(1*H*)-iminopyridines **8a** and **8b** were also prepared in fair yields by applying the aforementioned one pot reaction of the compound **1**, the corresponding aldehyde and malononitrile (Scheme 2, Table 1). The structures of the newly synthesized compounds were confirmed on the basis of their elemental analysis and IR, ^1^H-NMR, ^13^C-NMR and mass spectral data.

### 2.2. Anticancer Screening

All the newly synthesized compounds were tested at the Department of Tumor Pathology, National Cancer Institute, Cairo, Egypt. Two cell lines were used for the evaluation – human cervix carcinoma cell line (Hela) and human breast carcinoma cell line (MCF7). The newly synthesized analogs **3a-c**, **4**, **5a**,**b**, **6a,b**, **7a-c** and **8a,b** were tested for *in vitro* cytotoxic activity against these cell lines, which were obtained frozen in liquid nitrogen (-180 °C) from the American Type Culture Collection [26]. The tumor cell lines were maintained in the National Cancer Institute, Cairo, Egypt, by serial sub-culturing.

The results (Table 2) are expressed in the form of the concentration of compound that causes 50% inhibition of cells growth. The *in vitro* evaluation revealed that all the newly synthesized compounds showed certain activity against tumor cell lines tested, although the activity was generally higher towards the cervical cancer line than the breast cancer one. Compound **3a** showed potent and broad antitumor activity against the two tumor cell lines tested (IC_50_ = 3.5 against Hela and IC_50_ = 4.5 against MCF7) compared to the potent anticancer drug 5-flourouracil (5-FU) used as a reference standard [27]. Substitution of the side chain with different ring systems like in the oxopyridine or thioxopyridine compounds **6a**,**b** and **7a,b** resulted in moderate activity against the Hela cell line and marked activity against the MCF-7 cell line. Meanwhile, substitution of the side chain with iminopyridine, thioxopyrimidine and/or pyrazoline rings resulted in low activity against the two tumor cell lines. These results demonstrated that changing the molecular conformation and orientation could influence markedly the antitumor activity against the two tested cell types.

## 3. Experimental

### 3.1. General

Melting points (°C, uncorrected) were measured in open glass capillaries using a Barnstead 9001 electrothermal melting point apparatus. Infrared spectra (ν, cm^-1^) were recorded on a Jasco FT/IR-330E, Fourier Transform Infrared Spectrometer using KBr discs. ^1^H-NMR spectra were determined using a Bruker AC 500 Ultra Shield NMR spectrometer operating at 500.13 MHz for ^1^H and 125.76 MHz for ^13^C, the chemical shifts are expressed in δ (ppm) downfield from tetramethylsilane (TMS) used as internal standard. Electron impact mass spectra (EI-MS) were recorded on a Shimadzu GC-MS-QP 5000 instrument at 70 eV. Elemental analyses (C, H, N) were in full agreement with the proposed structures within ±0.4% of the theoretical values. Monitoring the reactions and checking the purity of the final products were carried out by thin layer chromatography (TLC) using silica gel precoated aluminum sheets (60 F_254_, Merck) and visualization with ultraviolet light (UV) at 365 and 254 nm.

### 3.2. 3-Aryl-1-(1,2,3,4-tetrahydronaphthalen-6-yl)prop-2-en-1-ones ***3a-c***

A mixture of 6-acetyltetraline **1** (4.9 g, 0.028 mol), the appropriate aldehyde **2a-c** (0.028 mol) and 10% ethanolic sodium hydroxide solution (15 mL) in ethanol (30 mL) was stirred for 12 h. The reaction mixture was then warmed at 40 °C for 10 min. and the separated precipitate was filtered off and recrystallized from ethanol.

*3-(2,6-Dichlorophenyl)-1-(1,2,3,4-tetrahydronaphthalen-6-yl)prop-2-en-1-ones* (**3a**): IR: 1690 (C=O), 1650 (C=C). ^1^H-NMR (DMSO-d_6_): δ 1.7-1.73 (m, 4H, tetralin CH_2_), 2.76-2.8 (m, 4H, tetralin CH_2_), 7.21 (d, 1H, CH=, *J* = 10.4 Hz), 7.54 (d, 1H, CH=, *J* = 10.4 Hz), 7.37-7.8 (m, 6H, Ar-H). ^13^C-NMR: δ 22 (tetralin CH_2_), 29 (tetralin CH_2_), 125.5 (CH=), 143.2 (CH=), 129.01, 129.25, 130.59, 130.83, 132.11, 134.37, 134.3, 135.07, 136.2, 137.3 (Ar-C), 188.5 (C=O). MS, *m/z* (rel. int.): 334 (M^+^, 0.3), 176 (50), 175 (75), 173 (100), 138 (5).

*3-(2,6-Difluorophenyl)-1-(1,2,3,4-tetrahydronaphthalen-6-yl)prop-2-en-1-ones* (**3b**): IR: 1651 (C=O), 1548 (C=C), ^1^H-NMR (DMSO-d_6_): δ 1.7 (m, 4H, tetralin CH_2_), 2.5 (m, 4H, tetralin CH_2_), 7.24 (d, 1H, CH=, *J* = 8.3 Hz), 7.84 (d, 1H, CH=, *J* = 8.3 Hz), 6.66-7.6 (m, 6H, Ar-H); ^13^C-NMR: δ 22 (tetralin CH_2_), 29 (tetralin CH_2_), 129.3 (CH=), 136.6 (CH=), 111.2, 112.3, 126.7, 129.9, 131.16, 134.26, 134.3, 135.9, 142.5, 157.3 (Ar-C), 186.9 (C=O). MS, *m/z* (rel. int.): 298 (M^+^, 0.2), 165 (19), 139 (2), 137 (100), 131 (3), 75 (5).

*3-(3-Ethoxy-4-hydroxyphenyl))-1-(1,2,3,4-tetrahydronaphthalen-6-yl)prop-2-en-1-ones* (**3c**): IR: 3420 (OH), 1634 (C=O) 1539 (C=C). ^1^H-NMR: δ 1.35 (t, 3H, CH_3_, *J* = 7.1 Hz), 1.70-1.73 (m, 4H, tetralin CH_2_), 2.5 (m, 4H, tetralin CH_2_), 4.08 (q, 2H, CH_2_, *J* = 11.05 Hz), 7.15 (d, 1H, CH=, *J* = 9.4 Hz), 7.40 (d, 1H, CH=, *J* = 9.4 Hz), 6.97-7.4 (m, 6H, Ar-H), 9.75 (s, 1H, OH). ^13^C-NMR: δ 14.49 (CH_3_), 22.2 (tetralin CH_2_), 28.8 (tetralin CH_2_), 125.7 (CH=), 147.2 (CH=), 111.8, 113.3, 115.6, 123.7, 126.2, 128.4, 129.8, 136.7, 137.01, 141.9, 149.8, 153.2 (Ar-C), 188.56 (C=O). MS, *m/z* (rel. int.): 174 (30), 166 (67), 159 (100), 137 (100), 131 (17).

### 3.3. 1-Acetyl-4-(2,6-difluorophenyl)-3-(1,2,3,4-tetrahydronaphthalen-6-yl)-2-pyrazoline ***4***

Hydrazine hydrate (99%, 1.0 mL) was added to a solution of compound **3b** (2.98 g, 0.01 mol) in acetic acid (30 mL) and the mixture was heated under reflux for 6 h. and the excess solvent was distilled *in vacuo*. Cold water (30 mL) was then added to the remaining residue and the separated crude product was filtered, washed with water, dried and crystallized from acetic acid. IR: 2880-2910 (tetralin CH_2_ & CH_3_), 1710 (C=O). ^1^H-NMR (DMSO-d_6_): δ 1.76-1.87 (m, 4H, tetralin CH_2_), 4.1 (dd, 2H, pyrazoline CH_2,_ *J* = 14.2 Hz), 2.15 (s, 3H, CH_3_), 2.75-2.8 (m, 4H, tetralin CH_2_), 6.5-6.9 (m, 1H, pyrazoline CH), 7.0-7.5 (m, 6H, Ar-H). ^13^C-NMR: δ 22.3 (tetralin CH_2_), 24.4 (CH_3_), 28.92 (tetralin CH_2_), 38.67 (pyrazoline CH_2_), 39.67 (pyrazoline CH), 111.8, 123.7, 126.2, 128.4, 129.8, 136.7, 137.01, 149.8, 141.9, 153.2 (Ar-C), 157.3 (imine C), 168.2 (C=O). MS *m/z* (rel. int.): 169 (72), 141 (40), 116 (20), 96 (46), 74 (100).

### 3.4. 4-Aryl-6-(1,2,3,4-tetrahydronaphthalen-6-yl)-1,2-dihydropyrimidine-2-thiones ***5a,b***

A solution of potassium hydroxide (1.7 g, 0.03 mol) in water (4 mL) was added to a mixture of the appropriate α,β-unsaturated ketone **3a**,**b** (0.01 mol) and thiourea (0.9 g, 0.012 mol), in ethanol (50 mL) and the mixture was heated under reflux for 24 h. The excess of solvent was then distilled off and the residue was acidified with dilute hydrochloric acid. The separated crude product was filtered, washed with water, dried and crystallized from aqueous ethanol.

*4-(2,6-Dichlorophenyl)-6-(1,2,3,4-tetrahydronaphthalen-6-yl)-1,2-dihydropyrimidine-2-thione* (**5a**): IR: 3400 (NH), 2888-2921 (tetralin CH_2_), 1050 (C=S). ^1^H-NMR (DMSO-d_6_): δ 1.67-1.7 (m, 4H, tetralin CH_2_), 2.49-2.52 (m, 4H, tetralin CH_2_), 3.55 (s, 1H, NH), 5.5 (s, 1H, pyrimidine CH), 6.6-7.1 (m, 6H, Ar-H). MS, *m/z* (rel. int.): 159 (100), 131 (19), 91 (40), 78 (60).

*4-(2,6-Difluorophenyl)-6-(1,2,3,4-tetrahydronaphthalen-6-yl)-1,2-dihydropyrimidine-2-thione* (**5b**): IR: 3400 (NH), 2880-2925 (tetralin CH_2_), 1050 (C=S). ^1^H-NMR (DMSO-d_6_): δ 1.65-1.66 (m, 4H, tetralin CH_2_), 2.4-2.5 (m, 4H, tetralin CH_2_), 3.5 (s, 1H, NH), 5.5 (s, 1H, pyrimidine CH), 6.57-7.14 (m, 6H, Ar-H). ^13^C-NMR: δ 22 (tetralin CH_2_), 29 (tetralin CH_2_), 104 (pyrimidine CH), 105.5, 111.2, 126.7, 129.6, 131.16, 134.26, 134.3, 135.9, 142.5, 157.3 (Ar-C), 185 (C=S). MS, *m/z* (rel. int.): 131 (8), 113 (2), 112 (4), 96 (31), 76 (7).

### 3.5. 4-Aryl-6-(1,2,3,4-tetrahydronaphthalen-6-yl)-2-oxo-1,2-dihydropyridine-3-carbonitriles ***6a,b***

A mixture of 6-acetyltetraline **1** (1.74 g, 0.01 mol), ethyl cyanoacetate (1.13 g, 0.01 mol), the appropriate aldehyde **2a**,**b**, and ammonium acetate (6.0 g), was heated in *n*-butanol (40 mL) under reflux for 3 h. On cooling, the separated yellow solid was filtered, washed with water and crystallized.

*4-(2,6-Dichlorophenyl)-6-(1,2,3,4-tetrahydronaphthalen-6-yl)-2-oxo-1,2-dihydropyridine-3-carbonitrile* (**6a**): IR: 3330 (NH), 2987-2910 (tetralin CH_2_), 2228 (CN), 1616 (C=O). ^1^H-NMR (CDCl_3_): δ 1.79-1.82 (m, 4H, tetralin CH_2_), 2.76-2.82 (m, 4H, tetralin CH_2_), 7.25-7.48 (m, 7H, Ar-H), 7.75 (s, 1H, NH). ^13^C-NMR: δ 22 (tetralin CH_2_), 28 (tetralin CH_2_), 114.4 (CN), 105.5, 123.5, 127.4, 127.7, 130.4, 132.7, 133.7, 127.8, 129.5, 138.4, 141.6, 151.5, 156.4, 165.5 (Ar-C & pyridine-C), 162.69 (C=O). MS, *m/z* (rel. int.): 398 (M^+^, 0.7), 205 (100), 174 (4), 148 (3), 131 (11), 91 (20), 65 (3).

*4-(2,6-Difluorophenyl)-6-(1,2,3,4-tetrahydronaphthalen-6-yl)-2-oxo-1,2-dihydropyridine-3-carbonitrile* (**6b**): IR: 3430 (NH), 2990-2995 (tetralin CH_2_), 2217 (CN), 1600 (C=O). ^1^H-NMR (CDCl_3_): δ 1.75-1.82 (m, 4H, tetralin CH_2_), 2.76-2.81 (m, 4H, tetralin CH_2_), 6.8-7.2 (m, 7H, Ar-H), 7.61 (s, 1H, NH). MS, *m/z* (rel. int.): 362 (M^+^, 0.5), 174 (26), 166 (100), 159 (100), 131 (4), 118 (5).

### 3.6. 4-Aryl-6-(1,2,3,4-tetrahydronaphthalen-6-yl)-2-thioxo-1,2-dihydropyridine-3-carbonitriles ***7a**-**c***

A mixture of 6-acetyltetraline **1** (1.74 g, 0.01 mol), 2-cyanothioacetamide (1 g, 0.01 mol), the appropriate aldehyde **2a-b**, and ammonium acetate (6.0 g), was heated under reflux in *n*-butanol (40 mL) for 3 h. On cooling, the separated yellow solid was filtered, washed with water and crystallized.

*4-(2,6-Dichlorophenyl)-6-(1,2,3,4-tetrahydronaphthalen-6-yl)-2-thioxo-1,2-dihydropyridine-3-carbonitrile* (**7a**): IR: 3450 (NH), 2988-2995 (tetralin CH_2_), 2240 (CN), 1340 & 1140 (C=S). ^1^H-NMR (DMSO-d_6_): δ 1.8-1.82 (m, 4H, tetralin CH_2_), 2.77-2.81 (m, 4H, tetralin CH_2_), 6.5 (s, 1H, NH), 7.2-7.6 (m, 7H, Ar-H & pyridine-H). ^13^C-NMR: δ 22 (tetralin CH_2_), 30 (tetralin CH_2_), 115.5 (CN), 105, 123.5, 127.4, 127.7, 130.4, 132.7, 133.7, 127.8, 129.5, 138.4, 141.6, 105.5, 151.5, 156.4 (Ar-C & pyridine-C), 164 (C=S). MS, *m/z* (rel. int.): 281 (20), 207 (35), 147 (23), 135 (32).

*4-(2,6-Difluorophenyl)-6-(1,2,3,4-tetrahydronaphthalen-6-yl)-2-thioxo-1,2-dihydropyridine-3-carbonitrile* (**7b**): IR: 3450 (NH), 2910-2985 (tetralin CH_2_), 2240 (CN), 1310 & 1090 (C=S). ^1^H-NMR (DMSO-d_6_): δ 1.73-1.8 (m, 4H, tetralin CH_2_), 2.74-2.8 (m, 4H, tetralin CH_2_), 5.18 (s, 1H, NH), 7.15-7.82 (m, 7H, Ar-H & pyridine-H). MS, *m/z* (rel. int.): 378 (M^+^, 2.7), 361 (2), 207 (16), 159 (29), 78 (75).

*4-(3-Ethoxy-4-hydroxyphenyl)-6-(1,2,3,4-tetrahydronaphthalen-6-yl)-2-thioxo-1,2-dihydropyridine-3-carbonitrile* (**7c**): IR: 3470 (NH), 3310 (OH), 2820-2995 (tetralin CH_2_, CH_3_, CH_2_), 2250 (CN), 1310 & 1110 (C=S). ^1^H-NMR (DMSO-d_6_): δ 1.33-1.35 (t, 3H, CH_3,_ *J* = 2.93 Hz), 1.73-1.8 (m, 4H, tetralin CH_2_), 2.76-2.8 (m, 4H, tetralin CH_2_), 4.11 (q, 2H, C***H***_2_CH_3,_ *J* = 2.93 Hz), 9.7 (s, 1H, NH), 7.0-7.6 (m, 7H, Ar-H & pyridine-H). ^13^C-NMR: δ 15.5 (CH_3_), 22 (tetralin CH_2_), 30 (tetralin CH_2_), 58.5 (***C***H_2_CH_3_), 115.5 (CN), 102.5, 104.0, 110.5, 129.4, 133.7, 135.4, 138.7, 140.7, 127.8, 128.5, 142.4, 145.6, 105.5, 153.5, 158.4, 166.5 (Ar-C & pyridine-C), 168 (C=S). MS, *m/z* (rel. int.): 174 (33), 159 (100), 131 (20), 104 (2).

### 3.7. 4-Aryl-6-(1,2,3,4-tetrahydronaphthalen-6-yl)-2-imino-1,2-dihydropyridine-3-carbonitriles ***8a**,**b***

A mixture of 6-acetyltetraline **1** (1.74 g, 0.01 mol), malononitrile (0.66 g, 0.01 mol), the appropriate aldehyde **2a-b**, and ammonium acetate (6.0 g), was heated under reflux in *n*-butanol (40 mL) for 3 h. On cooling, the separated yellow solid was filtered, washed with water and crystallized.

*4-(2,6-Dichlorophenyl)-6-(1,2,3,4-tetrahydronaphthalen-6-yl)-2-imino-1,2-dihydropyridine-3-carbonitrile* (**8a**): IR: 3500, 3450 (NH), 2820-2995 (tetralin CH_2_), 2250 (CN). ^1^H-NMR (CDCl_3_): δ 1.79-1.82 (m, 4H, tetralin CH_2_), 2.1 (s, 1H, NH), 2.81-2.83 (m, 4H, tetralin CH_2_), 5.61 (s, 1H, NH), 7-7.6 (m, 7H, Ar-H & pyridine-H). ^13^C-NMR: δ 22.2 (tetralin CH_2_), 28.7 (tetralin CH_2_), 114.9 (CN), 104.0 123.7, 127.4, 127.6, 128.8, 130.01, 133.2, 136.8, 149.9, 159.06, 110.9, 128.6, 159.9, 162.0, 166.5 (Ar-C & pyridine-C), 176.06 (***C***=NH). MS, *m/z* (rel. int.): 207 (100), 159 (98), 151 (8), 131 (15), 119 (3).

*4-(2,6-Difluorophenyl)-6-(1,2,3,4-tetrahydronaphthalen-6-yl)-2-imino-1,2-dihydropyridine-3-carbonitrile* (**8b**): IR: 3470, 3450 (NH), 2820-2990 (tetralin CH_2_), 2250 (CN). ^1^H-NMR (CDCl_3_): δ 1.81-1.826 (m, 4H, tetralin CH_2_), 2.1 (s, 1H, NH), 2.81-2.83 (m, 4H, tetralin CH_2_), 5.61 (s, 1H, NH), 6.9-7.67 (m, 7H, Ar-H & pyridine-H). ^13^C-NMR: δ 22.36 (tetralin CH_2_), 28.91 (tetralin CH_2_), 115.8 (CN), 104.5, 111.37, 111.68, 112.14, 123.98, 127.64, 129.15, 129.54, 131.03, 131.12, 137.23, 155.5, 1157.6, 161.3, 166.6 (Ar-C & pyridine-C), 174.1 (C=NH). MS, *m/z* (rel. int.): 194 (5), 131 (100), 127 (100), 104 (100).

### 3.8. Determination of the anticancer activity *[26]*

Cell monolayers were fixed with 10% (w/v) trichloroacetic acid and stained for 30 minutes with 0.4% (wt/vol) sulforhodamine B (SRB) dissolved in 1% acetic acid. Unbound dye was removed by four washes with 1% acetic acid, and protein-bound dye was extracted with 10 mM unbuffered Tris base [tris (hydroxymethyl) aminomethane] for determination of optical density in a computer-interfaced, 96-well microtiter plate reader at 510 nm.

## 4. Conclusions

New tetrahydronaphthaline derivatives bearing different heterocyclic moieties were tested for *in vitro* antitumor activity. The results indicated that the 2,6-dihaloarylchalcone derivatives **3a,b**, the cyanopyridone derivatives **6a,b** and thioxopyridine derivatives **7a-c** were the most effective against the cervix cell line (Hela), showing IC_50_ values of 3.5, 10.5, 7.1, 10.9, 8.1, 5.9 and 6.5 μg/mL, respectively. All the tested compounds showed moderate to marginal activity against the breast carcinoma cell line MCF-7. Compound **3a** was the most potent, with an IC_50_ value of 3.5 μg/mL against Hela and 4.5 μg/mL against MCF7 cell line. Compound **3a** seems a promising new lead compound with a novel skeleton for further development towards a new potential clinical trials candidate.

## Figures and Tables

**Table 1:** Melting points, crystallization solvents, yields, molecular formulae and molecular weights of newly synthesized compounds **3a-c**, **4**, **5a**,**b**, **6a**,**b**, **7a-c** and **8a**,**b**.

Comp. No.	X	Mp (ºC)	Cryst. Solv.	Yield (%)	Molecular Formula (Mol. Wt.)
**3a**	2,6-Cl_2_	71-3	EtOH	53	C_19_H_16_Cl_2_O (331.24)
**3b**	2,6-F_2_	250-2	EtOH	42	C_19_H_16_F_2_O (298.34)
**3c**	3-EtO,4-OH	77-9	EtOH	80	C_20_H_20_O_2_ (292.38)
**4**	-	> 300	AcOH	95	C_21_H_20_F_2_N_2_O (354.40)
**5a**	2,6-Cl_2_	> 300	EtOH/H_2_O	20	C_20_H_16_Cl_2_ N_2_S (387.33)
**5b**	2,6-F_2_	253-5	EtOH/H_2_O	28	C_20_H_16_F_2_ N_2_S (354.42)
**6a**	2,6-Cl_2_	252-4	AcOH	18	C_22_H_16_Cl_2_ N_2_O (395.29)
**6b**	2,6-F_2_	250-2	AcOH	30	C_22_H_16_F_2_ N_2_O (362.38)
**7a**	2,6-Cl_2_	150-2	AcOH	25	C_22_H_16_Cl_2_ N_2_S (411.36)
**7b**	2,6-F_2_	124-6	AcOH	53	C_22_H_16_F_2_ N_2_S (378.45)
**7c**	3-EtO-4-OH	226-8	AcOH	90	C_23_H_20_ N_2_OS (372.49)
**8a**	2,6-Cl_2_	193-5	AcOH	26	C_22_H_17_Cl_2_ N_3_ (394.31)
**8b**	2,6-F_2_	100-2	AcOH	34	C_22_H_17_F_2_ N_3_ (361.40)

**Table 2:** Effect of compounds **3a-c**, **4**, **5a**,**b**, **6a,b**, **7a-c** and **8a,b** on cervix carcinoma cell line (Hela) and breast carcinoma cell line (MCF7).

Compound No.	IC_50_(μg/mL)	
	Hela	MCF7
**3a**	3.5	4.5
**3b**	10.5	15
**3c**	12.5	18.3
**4**	11.3	19
**5a**	10.7	20.5
**5b**	11.9	17.3
**6a**	7.1	12
**6b**	10.9	17.5
**7a**	8.1	16
**7b**	5.9	12.5
**7c**	6.5	16
**8a**	12.1	22.3
**8b**	12.1	21.7
**5-FU**	-	3.5
